# Gene Network Exploration of Crosstalk between Apoptosis and Autophagy in Chronic Myelogenous Leukemia

**DOI:** 10.1155/2015/459840

**Published:** 2015-03-03

**Authors:** Fengfeng Wang, William C. S. Cho, Lawrence W. C. Chan, S. C. Cesar Wong, Nancy B. Y. Tsui, Parco M. Siu, S. P. Yip, Benjamin Y. M. Yung

**Affiliations:** ^1^Department of Health Technology and Informatics, Hong Kong Polytechnic University, Lee Shau Kee Building, Hung Hom, Kowloon, Hong Kong; ^2^Department of Clinical Oncology, Queen Elizabeth Hospital, 30 Gascoigne Road, Kowloon, Hong Kong

## Abstract

*Background*. Gene expression levels change to adapt the stress, such as starvation, toxin, and radiation. The changes are signals transmitted through molecular interactions, eventually leading to two cellular fates, apoptosis and autophagy. Due to genetic variations, the signals may not be effectively transmitted to modulate apoptotic and autophagic responses. Such aberrant modulation may lead to carcinogenesis and drug resistance. The balance between apoptosis and autophagy becomes very crucial in coping with the stress. Though there have been evidences illustrating the apoptosis-autophagy interplay, the underlying mechanism and the participation of the regulators including transcription factors (TFs) and microRNAs (miRNAs) remain unclear. *Results*. Gene network is a graphical illustration for exploring the functional linkages and the potential coordinate regulations of genes. Microarray dataset for the study of chronic myeloid leukemia was obtained from Gene Expression Omnibus. The expression profiles of those genes related to apoptosis and autophagy, including MCL1, BCL2, ATG, beclin-1, BAX, BAK, E2F, cMYC, PI3K, AKT, BAD, and LC3, were extracted from the dataset to construct the gene networks. *Conclusion*. The network analysis of these genes explored the underlying mechanisms and the roles of TFs and miRNAs for the crosstalk between apoptosis and autophagy.

## 1. Introduction

Apoptosis is a kind of programmed cell death, which plays a very important role in maintaining the adult tissue homeostasis and supporting the embryonic tissue remodeling [1]. Besides the proper cell development, external factors, such as nutrient deprivation, toxin, hypoxia, and radiation, trigger the mechanism of apoptosis by inducing cellular stress and subsequent signal transmission through molecular interactions. B-cell CLL/lymphoma 2 (BCL2) homologues have been extensively studied and experimentally validated as the key antiapoptotic and proapoptotic regulators that control the outer membrane permeability or integrity of mitochondria for the release of cytochrome c [2]. Among the antiapoptotic BCL2 homologues, BCL2 and BCL-XL can inhibit the formation of cytochrome c/Apaf-1/caspase-9 apoptosome by binding their unique BH4 domain to the C terminal of apoptotic peptidase activating factor 1 (Apaf-1) [3, 4]. The myeloid cell leukemia 1 (MCL1) is another antiapoptotic BCL2 homologue whose degradation in response to the stress through translation inhibition enhances the activation of apoptosis [2]. However, the whole apoptotic process cannot be controlled tightly by the high responsiveness of MCL1 without the commitment of the downstream proapoptotic regulators, such as BCL2-associated X protein (BAX) and BCL2-antagonist/killer (BAK). As proapoptotic BCL2 homologues, BAX and BAK form homooligomers within the mitochondrial membrane and breach its integrity, activating the caspases and apoptosis. These negative and positive regulations of apoptosis stop the division of damaged cells selectively and control a viable cell number to reduce the burden of nutritional supply.

Autophagy is a catabolic process responding to the stress induced by the above-mentioned external factors. Different from apoptosis, autophagy helps the cells to survive and maintain their functions by eliminating the damaged organelles and recycling the obsolete cytosol. These damaged or obsolete materials are contained by autophagosome and then fuse with a lysosome for bulk degradation. The autophagosome is double-membrane vesicle regulated by a set of autophagy-related (ATG) genes and nucleated by a protein complex of beclin-1 and phosphatidylinositol 3-kinase (PI3K) [5]. BCL2, as mentioned above, an antiapoptotic regulator, can also inhibit autophagy by binding to beclin-1 at the endoplasmic reticulum and its dissociation with beclin-1 is required for inducing autophagy [6]. In the same family, MCL1 regulates autophagy through its degradation under stress and interaction with beclin-1 on mitochondria [2]. However, the degradation of MCL1 or the inhibition of BCL2 is not decisive to activate autophagy without the fusion between autophagosomes and lysosomes regulated by lysosomal inhibitors and dissociation with ATG proteins [5, 6]. Thus, the interactions between the upstream and downstream molecules, such as beclin-1 and ATG, are also critical for the activation of autophagy.

The relationship between apoptosis and autophagy depends on the cellular context. As the mechanisms of apoptotic and autophagic responses share common pathways but mutually inhibit each other, the cells may adapt to the stress with a combination of these responses or in a mutually exclusive manner. The apoptotic response can be postponed or transformed to the autophagic one when the essential apoptotic proteins, such as BAX and BAK, are removed or inhibited [5]. Also, the long-lived differentiated nerve cells are more susceptible to autophagy than apoptosis to maintain homeostasis under stress [2]. Conversely, the inhibition of autophagy by the deletion of beclin-1 drives the cells towards apoptosis. The cells undergo apoptosis when beclin-1, ATG, or PI3K is inactivated to block the autophagy at an early stage, or when the lysosomal protein LAMP2 is depleted to block the fusion of autophagosomes and lysosomes at the late stage [5]. These evidences support the polarization between apoptosis and autophagy. On the other hand, apoptosis and autophagy share the common inducers, which are BCL2 homology-3 (BH3) only proteins, and the common stress mediators, including reactive oxygen species (ROS), free Ca^2+^ ions, and ceramide, as well as transcription factor p53, in their pathways. Thus, the concurrent triggers of both processes are allowed [5]. Therefore, the cell survival and death have to be balanced to maintain the normal cell functions and suppress carcinogenesis. The participation of transcription factors (TFs) and microRNAs (miRNAs) is crucial to tune the interplay by imposing changes in the expression of genes related to apoptosis and autophagy.

Transcriptional regulation is a kind of molecular interactions where the TF coded by a gene binds to a specific site in the 5′ untranslated region (UTR) of the target gene to regulate its expression [7]. As the change in the expression of a TF could be relayed to its target gene through such protein-DNA binding, transcriptional regulation may account for the coexpression of TF and target gene [8, 9]. By the same principle, a gene pair may exhibit correlated expression profiles when these two genes are concurrently regulated by a common TF [8]. The translation process of a gene is regulated by miRNAs, noncoding transcripts of approximately 21 nucleotides long. Through the imperfect base pairing with a binding site in the 3′ UTR of mRNA, miRNA regulates the expression of the target gene or destabilizes its mRNA [10]. It was shown that most of the miRNA-mRNA pairs exhibit highly correlated expression profiles, though both negative and positive correlations [11]. It is straightforward to anticipate the coexpression of two target genes, which are concurrently regulated by a common miRNA.

In a bioinformatics study, the gene-gene interactions controlling the human T helper cell differentiation process were identified by coexpression network but many of which would not be detected using differential expression [12]. Coexpressed genes tend to participate in the same regulatory and signaling circuits, forming complexes, pathways, and network modules [9, 13–16]. Further, strong coexpression was proved to cohere with higher gene ontology (GO) similarity and protein-protein interaction than that of random gene pairs [12].

This study adopted a gene network analysis approach based on coexpression measure. Correlation coefficient is a scale-invariant statistic that can be applied to measure the gene coexpression [17]. Two genes are linked if their correlation exceeds a specific threshold. Some existing approaches attempted to optimize the threshold with respect to the statistical significance of correlation or the network complexity, but not to the overall coexpression profiles of the disease and the normal states [12, 18]. Underlying mechanisms of gene interaction can be deciphered by contrasting the coexpression networks of the disease and the normal groups.

Chronic myelogenous leukemia (CML) is considered as the disease of interest for gene network analysis. The disrupted and invoked gene connections in CML represent the impaired mechanism when compared with the healthy individuals. In CML, a number of mitogenic signaling pathways, such as mitogen-activated protein kinase (MAPK) pathway and janus kinase (JAK)/signal transducer and activator of transcription (STAT) pathway, are activated so that the pluripotent hematopoietic stem cells aberrantly proliferate and differentiate to granulocytes in the blood [19]. The reciprocal translocation between chromosomes 9 and 22 results in the oncoprotein expressed from the BCR-ABL fusion oncogene and triggers the mitogenic signaling pathways. Apoptosis and adhesion properties of hematopoietic progenitors are deregulated by BCR-ABL, leading to massive leaving of immature progenitors from bone marrows [23]. The participation of autophagy and the transcriptional and posttranscriptional regulations in the molecular mechanism altering the progenitor cell functions is still unclear. This study is aimed to compare the CML patients with the healthy individuals in terms of the balance control between apoptosis and autophagy and identify the roles of TFs and miRNAs in the control mechanism. The gene networks provide global interactomic information to facilitate deeper understanding of carcinogenesis and identification of efficacious drug targets.

## 2. Methods

### 2.1. CML Dataset

The microarray dataset, analyzed in this work, was provided by a study comparing the normal and the CML hematopoietic stem/progenitor cells in gene expression [27]. The study recruited eight (Philadelphia) Ph+ CML patients and collected their peripheral blood. The bone marrows of four healthy donors were purchased from private sectors. The CD34+ cells were selected and sorted to G0 and G1/S/G2/M fractions. There were eventually 24 samples (16 CML and 8 normal) after the sorting. Total RNA was isolated from the cells of each sample, labeled, and hybridized to Human Genome U133 plus 2.0 arrays. The dataset has been deposited on the Gene Expression Omnibus (GEO) under the Accession number GSE24739 for public access.

Based on the relevance to apoptosis, autophagy, cell cycle, and CML, twenty genes were selected for the coexpression network analysis.  Table 1 shows the human genome organization (HUGO) gene symbols of the selected genes and the corresponding references supporting the relevance. This work considered a small portion of the related genes because it is aimed to differentiate the disease from the normal patterns of functional linkages between the key mediators and markers in molecular level.

### 2.2. Coexpression Measure

Coexpression between two genes can be quantified by a measure evaluating how similar their expression patterns are across the biological samples. The scale invariant property of Pearson correlation coefficient makes it a suitable choice for measuring the similarity between the expression patterns [13, 17]. Let *x*_*i*_ and *x*_*j*_ and cor(*x*_*i*_, *x*_*j*_) be the expression profiles of the *i*th and *j*th genes extracted from the expression matrix and the Pearson correlation coefficient between them. The coexpression level, *C*(*i*, *j*), was defined as in the formula (1) [17]:

(1) $$\begin{array}{c} C\left(i,j\right)=\left|\text{cor}\left(x_{i},x_{j}\right)\right|. \end{array}$$

The absolute value was taken because the coexpression measure will output a scalar in the range from 0 to 1 where a high output indicated a strong biological relationship in either positive or negative direction and a low output indicated a weak biological relationship. Such implementation ensured that the inhibiting molecular interactions, such as degradation of MCL1 by beta-TrCP [28], can be detected using this measure. The coexpression level was denoted by *C*_*d*_(*i*, *j*) if two expression profiles were extracted across samples of the disease (CML) group and *C*_*n*_(*i*, *j*) for the normal group.

### 2.3. Threshold Selection

In this study, a network presented genes as nodes and connected them with undirected edges if their coexpression levels exceeded a particular threshold value [12, 17]. In order to obtain two gene networks that characterized and differentiated the disease and the normal states, an optimal threshold of coexpression level was identified to classify the gene pairs in the disease and the normal states into strong and weak coexpression classes so that the classes were best associated with the groups. Two-sample Kolmogorov-Smirnov (KS) test was a good choice because it was sensitive to the differences in the distributions of two samples, that is, *C*_*d*_ and *C*_*n*_ in this case, and gave a threshold value, at which the deviation between the cumulative distribution functions of *C*_*d*_ and *C*_*n*_ was the maximum [29]. Let *F*_*d*_, *F*_*n*_, and *D* be the cumulative distribution functions (CDFs) of *C*_*d*_ and *C*_*n*_ and the maximum deviation, respectively. The value of *D* was given by the following formula (2):

(2) $$\begin{array}{c} D=\underset{C}{\max}\left|F_{d}\left(C\right)-F_{n}\left(C\right)\right|. \end{array}$$

Note that the inequalities inside the CDFs were inverted because our interest focused on the strong coexpression (Formula (3)). The optimal threshold (*C*) represented a coexpression level, at which *F*_*d*_ and *F*_*n*_ were extremely deviated. After the optimal threshold was identified, the gene pairs can be bisected into two coexpression classes. Chi-square test was also used to verify the association between the coexpression class and the disease. Consider

(3) $$\begin{array}{c} F_{d}\left(C\right)=\text{Prob}\left(C_{d}\geq C\right),\\ F_{n}\left(C\right)=\text{Prob}\left(C_{n}\geq C\right). \end{array}$$

### 2.4. Gene Network Construction

For clearer illustration of gene network, the identified gene pairs were classified into common, normal-specific, and disease-specific connections. The common connections were defined as the strongly coexpressed pairs shared by both the disease and the normal groups. The disease-specific connections, that is, CML-specific, were the strongly coexpressed pairs in the disease group with the common connections removed. The normal-specific connections were the strongly coexpressed pairs in the normal group with the common connections removed. Each type of connections can form a coexpression network having a particular biological meaning. The normal-specific connections were the potential molecular interactions maintaining physiological balance in healthy individuals. The disease-specific connections represented the characteristics of the disease.

A coexpression network consisted of genes connected by edges. Pajek was used to analyze and visualize the coexpression networks because it supports the global and local views of networks with various abstraction, visualization, and algorithmic tools [30]. Further, the coexpression levels were transformed and input along with the gene pairs to Pajek so that their values can be reflected by the edge thicknesses in the network. To display edges with thickness from 1 to 6 points, the coexpression levels between the threshold value and the maximum value were linearly transformed to the range from 1 to 6. Thus, the thicker edges could catch more attention in the visualization.

### 2.5. Identification of Regulatory Signatures

Composite regulatory signature database (CRSD) is a bioinformatic web-based resource, which integrates UniGene, mature miRNAs, putative promoter, TRANSFAC, pathway, GO, miRNA regulatory signature (MRS), and TF regulatory signature (TRS) databases to facilitate the comprehensive analysis of gene regulation networks [31]. MRS is defined as a set of interactions between a miRNA and a group of genes with its putative targets in the 3′ UTR. TRS is defined as a set of interactions between a TF and a group of genes with its putative binding sites in the 5′ UTR. Combining MRSs and TRSs of a common group of genes yields the composite regulatory signature (CRS). CRSD was used to query the MRS, TRS, or CRS for the strongly coexpressed gene pairs. The identified signatures can help to explore how the TFs and miRNAs drive the normal-specific, disease-specific, and common gene coexpression patterns.

## 3. Results

### 3.1. Thresholds of Coexpression Levels

**Figure 1:**
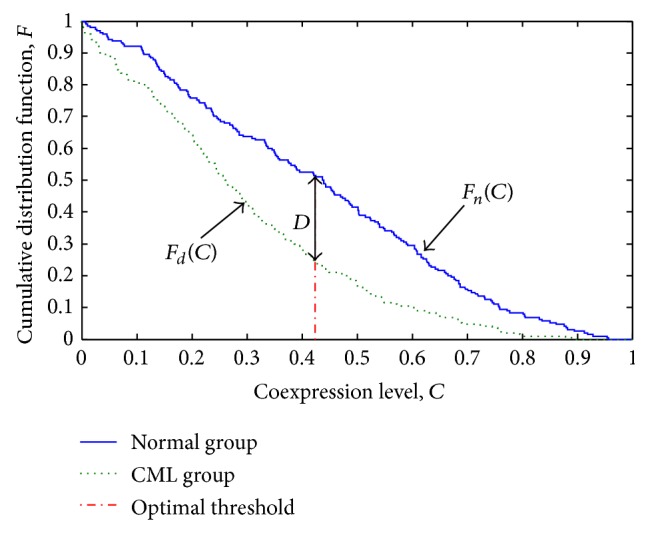
Cumulative distribution functions of coexpression levels for the normal and the CML groups with the candidate thresholds from 0 to 1.

Among 20 genes considered in this work (see Table S1 in Supplementary Material available online at http://dx.doi.org/10.1155/2015/459840), the coexpression levels of 190 gene pairs were computed for the normal group and the CML group independently. Gene pairs can be dichotomized into strong and weak coexpression classes, which characterized the corresponding groups. The threshold for the dichotomy was determined by two-sample KS test. The CDFs for the normal and the CML groups were numerically evaluated at every possible threshold value from 0 to 1 (Figure 1). It was found that the evaluated cumulative fractions were optimally deviated by *D*, 0.2789, at the coexpression level *C*, 0.4233 (optimal threshold). The KS test indicated that the two distributions were significantly different (*P* value < 0.01 for the statistic *D* = 0.2789). The contingency table of the gene pair counts at the optimal threshold is shown in Table 2. At the optimal threshold, the dichotomy of gene pairs was significantly associated with the disease as the Chi-square statistic was 31.4957 (*P* value < 0.01). The differential coexpression distribution suggested that the genes related to apoptosis and autophagy, in overall, exhibited more robust functional links in the normal group than the CML group.

### 3.2. Gene Networks

**Figure 2:**
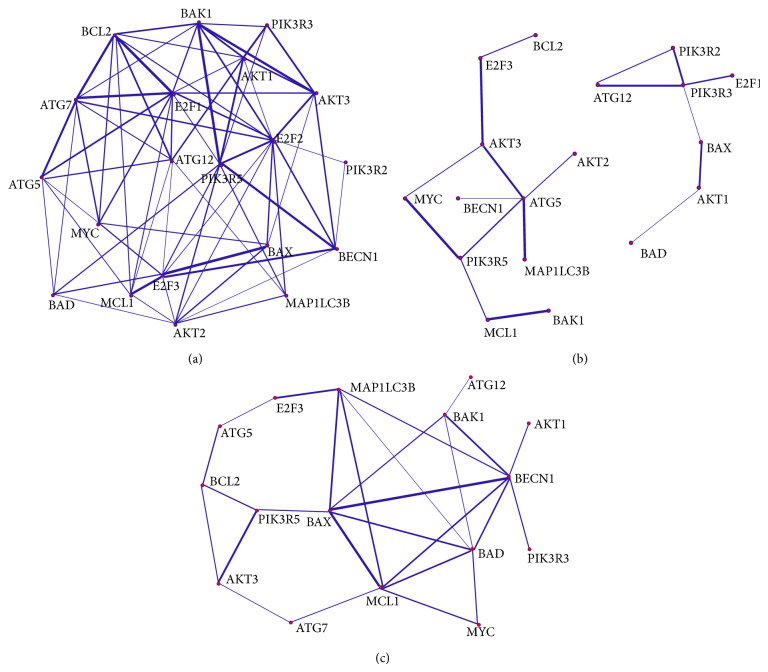
Coexpression networks (Using Pajek). The nodes represent genes. The edges indicate the strong correlation between nodes. The edge thickness reflects the coexpression levels. (a) Normal-specific coexpression network. (b) CML-specific coexpression network. (c) Common coexpression network.

The CML and the normal groups shared 27 common strongly coexpressed gene pairs according to the optimal threshold. After removing the common gene pairs from the strong coexpression class, the normal-specific gene pairs comprised 71 pairs and the CML-specific comprised 18 pairs. The coexpression networks for the normal-specific, CML-specific, and common gene pairs were constructed as shown in Figure 2.

### 3.3. Regulatory Signatures

**Figure 3:**
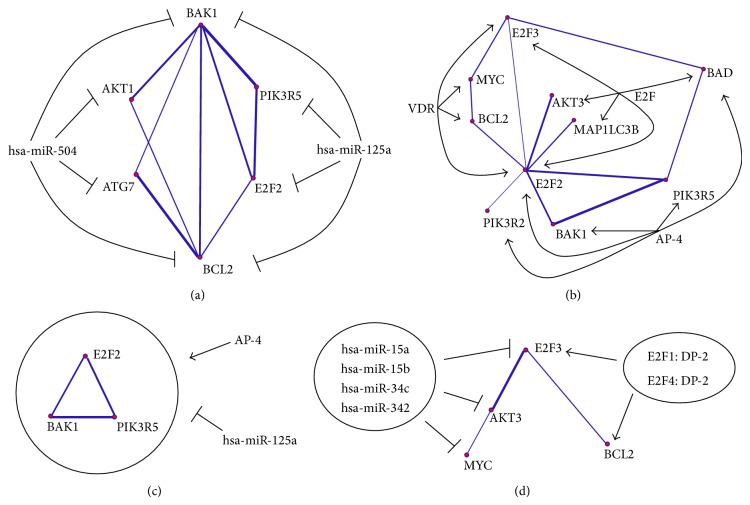
Regulatory signature (RS) networks. (a) Normal-specific MRS network. (b) Normal-specific TRS network. (c) Normal-specific CRS network. (d) CML-specific E2F3-RS network.

By querying the regulatory signatures in CRSD, the miRNAs and TFs predicted to target the normal-specific and the CML-specific gene pairs were identified. To maintain the significance of the identified regulatory signatures, a miRNA or TF was selected for further investigation if it targeted no less than four gene pairs. It was found that each of hsa-miR-504 and hsa-miR-125a concurrently regulates the expression of four genes in the normal-specific coexpression network, forming two MRSs. As the two MRSs shared two common genes, BAK1 and BCL2, they were combined to form the normal-specific MRS network (Figure 3(a)). It was also found that each of zinc finger protein (AP-4) and E2F concurrently regulates the expression of five genes, and vitamin D (1,25-dihydroxyvitamin D3) receptor (VDR) concurrently regulates the expression of four genes in the normal-specific coexpression network. As the three TRSs shared E2F2 as the common target gene, they were combined to form the normal-specific TRS network (Figure 3(b)). The connections coincided in the normal-specific MRS and TRS networks were the triangle linking E2F2, BAK1, and PIK3R5. These all three connections had strong coexpression levels and formed the normal-specific CRS network with AP-4 and hsa-miR-125a (Figure 3(c)).

In the CML-specific network, each of the identified miRNAs and TFs targets not more than two gene pairs. The MRS and TRS were not considered for further investigation because they were not so informative to suggest the concurrent regulations. Instead, it is interesting to note that E2F3 was linked to v-akt murine thymoma viral oncogene homolog 3 (AKT3) directly and v-myc avian myelocytomatosis viral oncogene homolog (MYC) indirectly, and these three genes are the predicted targets of hsa-miR-15a, hsa-miR-15b, hsa-miR-34c, and hsa-miR-342. Further, E2F3 and BCL2 were found to be strongly coexpressed, which are the predicted targets of E2F1:DP-2 and E2F4:DP-2. Among these genes, E2F3 is predicted to be coordinately regulated by four miRNAs and two TFs. These connections were combined to form the CML-specific E2F3 regulatory signature (E2F3-RS) network (Figure 3(d)).

## 4. Discussion

### 4.1. Disease-Associated Coexpression Threshold

Protein encoded by a gene performs its functions through the molecular interactions with that of the other genes. Without considering its functional partners, the expression level and differential expression of a gene are not informative enough to indicate whether it performs its known functions. Coexpression level between two genes quantifies the extent, in which the change in expression level of a gene coincides with that of the other. There may not be a coexpression threshold that can indicate the molecular interactions of two genes, but a threshold exists for identifying the strongly coexpressed gene pairs to optimally differentiate the normal and the disease groups in terms of the functional linkages. In our study, more strongly coexpressed gene pairs were found in the normal group than the CML group (Table 2). It implied that many functional links between genes, which may react to the external factors to further maintain the proper cellular functions or the tissue homeostasis in the normal group, were impaired in the CML group. The impaired connections may provide useful information for understanding the underlying molecular interaction mechanism and exploring the novel drug targets of CML.

### 4.2. Functional Coexpression Patterns

Genes highly connected with other genes act as the hubs for relaying the adaptive changes in gene expression through the molecular interactions. In the normal-specific network (Figure 2(a)), E2F1, E2F2, and E2F3 established, respectively, 11, 13, and 9 connections with other genes, implicating their central roles in the proper regulation of apoptosis, autophagy, and cell cycle. E2F1, E2F2, and E2F3 activate the cell cycle progression and drive the cells from quiescent (G0) into synthesis (S) phase [32]. The interplay of E2F1, E2F2, and E2F3 with other genes in the network maintained the balance between cell death, survival, and proliferation. Three remarkable coexpression patterns connected by E2F1, E2F2, and E2F3 were found in the normal-specific network and further discussed as follows.

In the normal-specific network, E2F2 was connected with BAK1, PIK3R5, and AKT3 in the high coexpression levels (correlation coefficients were 0.701, 0.778, and −0.756, resp.). BAK1 is a proapoptotic molecule. The positive correlation implied that when E2F2 was upregulated to speed up the cell cycle for the cellular proliferation, BAK1 may respond with the upregulation to promote apoptosis to control the cell number. We also revealed that PIK3R5 was an essential hub with 10 connections in the network. PIK3R5 is a regulatory subunit, which combines with a catalytic subunit to form the class I PI 3-kinase (PI3K). Since the PI3K pathway contributes to antiapoptosis and cell survival, E2F2 and PIK3R5 may be activated by RAS to promote the proliferation in the normal group [33]. In the common coexpression network (Figure 2(c)), PIK3R5 and AKT3 were strongly coexpressed in both two groups, but the correlation coefficients for the normal and the CML groups were of opposite signs (normal: −0.802; CML: 0.757). It implicated that the activation of AKT3 by PI3K may be retarded by the 3′-phosphoinositide phosphatase (PTEN) in the normal group so that the growth signal could not be relayed to mTOR signaling pathway and thus autophagy was possibly allowed [6, 34]. In contrast, the PI3K/AKT signaling may repress autophagic response in the CML group so that the damaged organelles could not be degraded. The negative correlation between E2F2 and AKT3 implied that the proper autophagy could be maintained during the cell cycle progression in the normal group. From the above observations, we can hypothesize that E2F2 favors both apoptosis and autophagy.

The normal-specific network showed that E2F1 was connected with ATG5, ATG7, ATG12, BCL2, and MYC in the high coexpression levels (correlation coefficients were −0.708, 0.883, −0.627, 0.954, and 0.642, resp.). ATG5 and ATG12 are involved in the vesicle elongation of the autophagy, while ATG7 helps the covalent conjugation of ATG5 and ATG12 [5]. During the cell proliferation, autophagy may be partially inhibited, as ATG5 and ATG12 are downregulated at the early stage of autophagy, but their covalent conjugation is readily facilitated by the upregulation of ATG7. BCL2 inhibited the apoptosis during cell cycle progression as its expression was positively correlated with E2F1 in the normal group. The positive correlation between E2F1 and MYC is supported by the mutual induction of gene expression [35]. MYC is a proapoptotic molecule. The antiapoptotic and proapoptotic responses of BCL2 and MYC coexisted in the normal-specific network because they can activate different pathways. BCL2 regulates the release of cytochrome c and caspase activation and then inhibits apoptosis [1–4]. MYC triggers the p53 signaling pathway to induce cell death when DNA damage happens [36]. It is hypothesized that E2F1 linking to various genes can promote and inhibit apoptosis through different pathways and partially links to autophagy.

It was shown in the normal-specific network that MCL1, BAX, and beclin-1 (BECN1) were connected with E2F3 in the high coexpression levels (correlation coefficients were −0.876, −0.957, and −0.804, resp.). BAX and BECN1 can promote apoptosis and autophagy, respectively, [1, 3, 5]. The negative correlation implied that both apoptosis and autophagy were inhibited when E2F3 was upregulated during the cell proliferation. MCL1 expression was also negatively correlated with E2F3. The hematopoietic cells may not be so sensitive to the stress as MCL1, a stress sensor, was downregulated during the cell cycle progression. Conversely, these three genes were upregulated to promote autophagy and apoptosis when the cells were situated in the G0 phase. In contrast, MCL1, BAX, and BECN1 were strongly coexpressed with each other without the participation of E2F3 in the common coexpression network (correlation coefficients of BECN1 and MCL1, BAX and MCL1, and BAX and BECN1 were 0.607, 0.831, and 0.895, resp.). The results demonstrated the persistent interplay between apoptosis and autophagy. In all, we can hypothesize that E2F3 opposes against both apoptosis and autophagy.

In the CML-specific network (Figure 2(b)), E2F3 and AKT3 were connected with a positive correlation (0.660). Though E2F3 opposed autophagy again as in the normal group, AKT3 responded to oncogenic or endoplasmic reticulum stress that were different from the stress detected by MCL1 [34].

### 4.3. Regulatory Mechanisms

The hsa-miR-504 and hsa-miR-125a MRSs shared the BAK1 and BCL2 connection as a common link in the normal-specific MRS network (Figure 3(a)). The balance between the proapoptotic and antiapoptotic properties of BAK1 and BCL2 was supported by their positive correlation (0.696), which may be induced by the coordinate regulation of hsa-miR-504 and hsa-miR-125a.

Three normal-specific TRSs were observed where VDR, E2F, and AP-4 are predicted as the TFs (Figure 3(b)). Again, E2F2 was the hub at the center of the TRSs and concurrently targeted by the three TFs. The E2F TRS concurred with the autoregulatory mechanism of E2F family proteins in cell cycle regulation. It was illustrated that the gene silencing of AP-4 is able to trigger apoptosis [37]. Apoptotic regulatory roles of AP-4 were further justified by the fact that the genes related to apoptosis, BAK1, BAD, PIK3R2, and PIK3R5, are predicted to be its targets.

It was straightforward to observe a motif shared by the MRS and TRS networks, that is, the CRS consisting of E2F2, BAK1, and PIK3R5 (Figure 3(c)). The correlation coefficients between them were positive and high (E2F2 and BAK1: 0.701; E2F2 and PIK3R5: 0.778; BAK1 and PIK3R5: 0.913). Through the coregulation by AP-4 and hsa-miR-125a, these three genes established a tight balance between cell death and survival when the cell proliferation was activated.

In Figure 3(d), E2F3 is predicted as the common target of four miRNAs and two TFs. The TFs and miRNAs were found to be counteracted to control the expression level of E2F3. It was proved that the deletion of miR-15 was frequently found in chronic lymphocytic leukemia (CLL) [38]. It is anticipated that the overexpression of E2F3 caused by the deletion of miR-15 may induce myeloid malignancy. Further, the miRNAs, including miR-15a, downregulated both E2F3 and AKT3 and maintained their strong coexpression in the CML group. These evidences further justify that E2F3-AKT3 connection may be CML-specific.

## 5. Conclusion

Gene network analysis helps us to explore the gene connectivity and the potential functional linkages. This work adopted an approach for identifying the gene pairs with strong coexpression classified by a disease-associated threshold. CML was the disease of interest in this work. The normal-specific network illustrated the gene connections found in the proper cellular regulation but not in cancer molecular mechanism. As the key transcription factors of cell cycle regulation, E2F1, E2F2, and E2F3, acted as the hubs for the normal-specific connections. E2F1 was associated with antiapoptosis and proapoptosis through different pathways but partially associated with autophagy. E2F2 was linked with the promotion of apoptosis and autophagy, while E2F3 exhibited opposition to apoptosis and autophagy. In the CML-specific network, the link between E2F3 and AKT3 demonstrated a possible cellular response to oncogenic stress in the proliferation of hematopoietic cells. It is important to note that E2F3 and AKT3 are both the predicted targets of miR-15, whose deletion was proved to be associated with cancer. The coregulations of genes by miRNAs and TFs were indicated by the MRS, TRS, and CRS. The central role of E2F2 was further confirmed by the normal-specific TRS network. In the normal-specific MRS network, the apoptotic balance was strengthened by the coregulation of BAK1 and BCL2 by miRNAs. As a normal-specific CRS, the E2F2-BAK1-PIK3R5 motif may constitute the core mechanism controlling the cell cycle progression, apoptosis, and autophagy, which requires further investigation in the future experimental studies.

## Supplementary Material

Supplementary Table: Twenty candidate genes for gene network analysis are shown in Table S1.

## Figures and Tables

**Table 1:** List of selected genes, relevance, and supporting references.

HUGO gene symbol	Relevance	References
MCL1; BCL2; BAD	Apoptosis; autophagy; CML	[2, 19, 20]
BAX; BAK1	Apoptosis	[1–3, 6]
E2F1; E2F2; E2F3; MYC	Apoptosis; autophagy; cell cycle; CML	[3, 6, 19, 21, 22]
PIK3R2; PIK3R3; PIK3R5; AKT1; AKT2; AKT3	Apoptosis; cell cycle; CML	[19, 23–25]
ATG5; ATG7; ATG12; MAP1LC3B; BECN1	Autophagy	[2, 5, 6, 26]

**Table 2:** Contingency table of gene pair counts at the optimal threshold.

Class	CML	Normal
Strong coexpression	45	98
Weak coexpression	145	92
