# Supplementary material for: Gene Network Exploration of Crosstalk between Apoptosis and Autophagy in Chronic Myelogenous Leukemia
Source: Biomed Res Int. 2015 Mar 3;2015:459840. doi: 10.1155/2015/459840 (PMC4363532; doi:10.1155/2015/459840)
Supplement: Supplementary file 1 — Supplementary Table: Twenty candidate genes for gene network analysis are shown in Table S1. [file 459840.f1.doc]

Table S1.Twenty candidate genes for gene network analysis.

| HUGO gene symbol | Gene title |
| --- | --- |
| AKT1 | v-akt murine thymoma viral oncogene homolog 1 |
| AKT2 | v-akt murine thymoma viral oncogene homolog 2 |
| AKT3 | v-akt murine thymoma viral oncogene homolog 3 |
| ATG12 | autophagy related 12 |
| ATG5 | autophagy related 5 |
| ATG7 | autophagy related 7 |
| BAD | BCL2-associated agonist of cell death |
| BAK1 | BCL2-antagonist/killer 1 |
| BAX | BCL2-associated X protein |
| BCL2 | B-cell CLL/lymphoma 2 |
| BECN1 | beclin 1, autophagy related |
| E2F1 | E2F transcription factor 1 |
| E2F2 | E2F transcription factor 2 |
| E2F3 | E2F transcription factor 3 |
| MAP1LC3B | microtubule-associated protein 1 light chain 3 beta |
| MCL1 | myeloid cell leukemia 1 |
| MYC | v-myc avian myelocytomatosis viral oncogene homolog |
| PIK3R2 | phosphoinositide-3-kinase, regulatory subunit 2 (beta) |
| PIK3R3 | phosphoinositide-3-kinase, regulatory subunit 3 (gamma) |
| PIK3R5 | phosphoinositide-3-kinase, regulatory subunit 5 |
